# Comparative study of four sigmoid models of pressure-volume curve in acute lung injury

**DOI:** 10.1186/1475-925X-6-7

**Published:** 2007-02-14

**Authors:** Guillermo M Albaiceta, Esteban Garcia, Francisco Taboada

**Affiliations:** 1 Intensive Care Unit, Hospital Universitario Central de Asturias, Oviedo, Spain; 2 Department of Functional Biology, University of Oviedo, Oviedo, Spain; 3 Department of Medicine, University of Oviedo, Oviedo, Spain

## Abstract

**Background:**

The pressure-volume curve of the respiratory system is a tool to monitor and set mechanical ventilation in acute lung injury. Mathematical models of the static pressure-volume curve of the respiratory system have been proposed to overcome the inter- and intra-observer variability derived from eye-fitting. However, different models have not been compared.

**Methods:**

The goodness-of-fit and the values of derived parameters (upper asymptote, maximum compliance and points of maximum curvature) in four sigmoid models were compared, using pressure-volume data from 30 mechanically ventilated patients during the early phase of acute lung injury.

**Results:**

All models showed an excellent goodness-of-fit (R^2 ^always above 0.92). There were significant differences between the models in the parameters derived from the inspiratory limb, but not in those derived from the expiratory limb of the curve. The within-case standard deviations of the pressures at the points of maximum curvature ranged from 2.33 to 6.08 cmH_2_O.

**Conclusion:**

There are substantial variabilities in relevant parameters obtained from the four different models of the static pressure-volume curve of the respiratory system.

## Background

The static pressure-volume curve is one of the main measurements on lung mechanics [1]. It is constructed by plotting the airway, transpulmonary or pleural pressure against lung volume in static conditions, this is, in the absence of gas flow. Values of pressure and volume define two limbs in the curve, depending on whether the data was acquired during stepwise increments or decrements in pressure and volume. These inspiratory and expiratory limbs are usually different due to the respiratory system hysteresis. The pressure-volume curves have been widely studied in acute lung injury and the acute respiratory distress syndrome [2]. They provide an assessment about the respiratory system mechanics, and their use has been proposed as a tool to set the mechanical ventilator [3].

Relevant data derived from the pressure-volume curve include total lung capacity, the maximal compliance and the value of the points of maximum curvature (which have been incorrectly cited in medical literature as "inflection points") of both limbs of the curve. There are different methods to determine these parameters [4]. Manual methods are based on the visual identification of three linear zones of the curve, defining the points as the intersections between these three lines, the maximal compliance as the slope of the medial section and total lung capacity as the maximum value reached. However, this eye-fitting has important interobserver variability [4,5]. To overcome this limitation, pressure-volume data can be fitted to mathematical models and the parameters calculated in an objective manner. Although different equations have been proposed for modelling pressure-volume curves, the sigmoid models [6-9] have shown an excellent fitting in experimental and clinical fields. However, some models have not been tested with clinical data, and the different models have not been compared.

### Hypothesis and objectives

The objective of this study was to assess the goodness of fit of four previously published sigmoid models of pressure-volume curve using data from patients with acute lung injury, and to compare the values of the calculated parameters. Our hypothesis was that the different models would yield similar results.

## Methods

Data from 30 patients (age 60 years [SD 14], APACHE-II score 24 [SD 6] [10], lung injury score 3.04 [SD 0.29] [11], PaO_2_/FiO_2 _146 mmHg [SD 43]) were retrospectively analyzed. Some data of these patients were previously published [12,13] Informed consent was obtained for each patient's next of kin.

All patients were studied during the first 72 hours after meeting acute lung injury criteria [14]. Exclusion criteria were: Less than 18 years old, contraindications to hypercapnia or elevated intrathoracic pressures (i.e. brain injury), air leaks, more than 5 days of mechanical ventilation or known or suspected chronic respiratory diseases. They were kept under deep sedation and muscle paralysis during the procedure, and ventilated in volume-controlled mode using an Evita 4 ventilator (Drager, Lubeck, Germany).

### Pressure-volume curve maneuver

Pressure and flow were measured using the ventilator built-in heated-wire pneumotachograph and pressure transducer, connected to the patient via an endotracheal tube. Volume was calculated by integration of flow. Data was acquired and stored in a computer via serial port, at a sampling rate of 125 Hz, using the VentView software (Drager, Lubeck, Germany). Before the maneuver, the system was checked for air leaks during a prolonged inspiratory pause.

All pressure-volume curves were traced using changes in a Continuous Positive Airway Pressure (CPAP) [5], as described in detail in previously published papers [12,13]. After three sighs (tidal volume 10 ml/Kg predicted body weight) to standardize volume history, the ventilator was switched to the CPAP mode, with a pressure level of 0 cmH_2_O. This pressure level was raised from 0 to 35 cmH_2_O in 5 cmH_2_O steps. After restoring basal ventilation for 3–5 minutes, three sighs more were applied and the ventilator switched again to CPAP mode at 0 cmH_2_O, allowing the complete deflation of the lungs. After this, pressure was raised to 35 cmH_2_O, and then decreased in 5 cmH_2_O steps to 0 cmH_2_O. Each pressure change was done only after reaching static conditions (zero flow). Changes in lung volume were computed for each pressure change. Data pairs of pressure and volume during the pressure increments were used to trace the inspiratory limb, and those from the decremental pressure maneuver used to trace the expiratory limb of the static pressure-volume curve.

### Mathematical models of the curve

Four mathematical models were studied [6-9]. All are sigmoid equations where volume and pressure are the dependent and independent variable respectively. Their equations and parameters are presented in Table 1.

**Table 1:** Equations for the mathematical models studied, and the general terms of lower and upper asymptotes, inflection point and points of maximum curvature. *The second derivative of model 3 is a transcendental equation, so a general term cannot be calculated.

			Volume		Pressure		
Model	Ref.	Equation	Lower asymptote	Upper asymptote	Inflection	LPMC	UPMC

1	21	V=a+b1+e−(P−c)/d MathType@MTEF@5@5@+=feaafiart1ev1aaatCvAUfKttLearuWrP9MDH5MBPbIqV92AaeXatLxBI9gBaebbnrfifHhDYfgasaacH8akY=wiFfYdH8Gipec8Eeeu0xXdbba9frFj0=OqFfea0dXdd9vqai=hGuQ8kuc9pgc9s8qqaq=dirpe0xb9q8qiLsFr0=vr0=vr0dc8meaabaqaciaacaGaaeqabaqabeGadaaakeaacqWGwbGvcqGH9aqpcqWGHbqycqGHRaWkdaWcaaqaaiabdkgaIbqaaiabigdaXiabgUcaRiabdwgaLnaaCaaaleqabaGaeyOeI0IaeiikaGIaemiuaaLaeyOeI0Iaem4yamMaeiykaKIaei4la8Iaemizaqgaaaaaaaa@3DFE@	*a*	*a *+ *b*	*c*	*c *- 1.317*d*	*c *+ 1.317*d*
2	18	V=b1+e−(P−c)/d MathType@MTEF@5@5@+=feaafiart1ev1aaatCvAUfKttLearuWrP9MDH5MBPbIqV92AaeXatLxBI9gBaebbnrfifHhDYfgasaacH8akY=wiFfYdH8Gipec8Eeeu0xXdbba9frFj0=OqFfea0dXdd9vqai=hGuQ8kuc9pgc9s8qqaq=dirpe0xb9q8qiLsFr0=vr0=vr0dc8meaabaqaciaacaGaaeqabaqabeGadaaakeaacqWGwbGvcqGH9aqpdaWcaaqaaiabdkgaIbqaaiabigdaXiabgUcaRiabdwgaLnaaCaaaleqabaGaeyOeI0IaeiikaGIaemiuaaLaeyOeI0Iaem4yamMaeiykaKIaei4la8Iaemizaqgaaaaaaaa@3BD1@	0	*b*	*c*	*c *- 1.317*d*	*c *+ 1.317*d*
3	12	V=V0−V0e−kP1+e−(P−c)/d MathType@MTEF@5@5@+=feaafiart1ev1aaatCvAUfKttLearuWrP9MDH5MBPbIqV92AaeXatLxBI9gBaebbnrfifHhDYfgasaacH8akY=wiFfYdH8Gipec8Eeeu0xXdbba9frFj0=OqFfea0dXdd9vqai=hGuQ8kuc9pgc9s8qqaq=dirpe0xb9q8qiLsFr0=vr0=vr0dc8meaabaqaciaacaGaaeqabaqabeGadaaakeaacqWGwbGvcqGH9aqpdaWcaaqaaiabdAfawnaaBaaaleaacqaIWaamaeqaaOGaeyOeI0IaemOvay1aaSbaaSqaaiabicdaWaqabaGccqWGLbqzdaahaaWcbeqaaiabgkHiTiabdUgaRjabdcfaqbaaaOqaaiabigdaXiabgUcaRiabdwgaLnaaCaaaleqabaGaeyOeI0IaeiikaGIaemiuaaLaeyOeI0Iaem4yamMaeiykaKIaei4la8Iaemizaqgaaaaaaaa@4522@	0	*V*_0_	*	*	*
4	11	V=a+b[1+e−(P−c)/d]s MathType@MTEF@5@5@+=feaafiart1ev1aaatCvAUfKttLearuWrP9MDH5MBPbIqV92AaeXatLxBI9gBaebbnrfifHhDYfgasaacH8akY=wiFfYdH8Gipec8Eeeu0xXdbba9frFj0=OqFfea0dXdd9vqai=hGuQ8kuc9pgc9s8qqaq=dirpe0xb9q8qiLsFr0=vr0=vr0dc8meaabaqaciaacaGaaeqabaqabeGadaaakeaacqWGwbGvcqGH9aqpcqWGHbqycqGHRaWkdaWcaaqaaiabdkgaIbqaamaadmaabaGaeGymaeJaey4kaSIaemyzau2aaWbaaSqabeaacqGHsislcqGGOaakcqWGqbaucqGHsislcqWGJbWycqGGPaqkcqGGVaWlcqWGKbazaaaakiaawUfacaGLDbaadaahaaWcbeqaaiabdohaZbaaaaaaaa@4196@	*a*	*a *+ *b*	c−dln⁡1s MathType@MTEF@5@5@+=feaafiart1ev1aaatCvAUfKttLearuWrP9MDH5MBPbIqV92AaeXatLxBI9gBaebbnrfifHhDYfgasaacH8akY=wiFfYdH8Gipec8Eeeu0xXdbba9frFj0=OqFfea0dXdd9vqai=hGuQ8kuc9pgc9s8qqaq=dirpe0xb9q8qiLsFr0=vr0=vr0dc8meaabaqaciaacaGaaeqabaqabeGadaaakeaacqWGJbWycqGHsislcqWGKbazcyGGSbaBcqGGUbGBdaWcaaqaaiabigdaXaqaaiabdohaZbaaaaa@356E@	c−dln⁡(3s+1)−5s2+6s+12s2 MathType@MTEF@5@5@+=feaafiart1ev1aaatCvAUfKttLearuWrP9MDH5MBPbIqV92AaeXatLxBI9gBaebbnrfifHhDYfgasaacH8akY=wiFfYdH8Gipec8Eeeu0xXdbba9frFj0=OqFfea0dXdd9vqai=hGuQ8kuc9pgc9s8qqaq=dirpe0xb9q8qiLsFr0=vr0=vr0dc8meaabaqaciaacaGaaeqabaqabeGadaaakeaacqWGJbWycqGHsislcqWGKbazcyGGSbaBcqGGUbGBdaWcaaqaaiabcIcaOiabiodaZiabdohaZjabgUcaRiabigdaXiabcMcaPiabgkHiTmaakaaabaGaeGynauJaem4Cam3aaWbaaSqabeaacqaIYaGmaaGccqGHRaWkcqaI2aGncqWGZbWCcqGHRaWkcqaIXaqmaSqabaaakeaacqaIYaGmcqWGZbWCdaahaaWcbeqaaiabikdaYaaaaaaaaa@4635@	c−dln⁡(3s+1)+5s2+6s+12s2 MathType@MTEF@5@5@+=feaafiart1ev1aaatCvAUfKttLearuWrP9MDH5MBPbIqV92AaeXatLxBI9gBaebbnrfifHhDYfgasaacH8akY=wiFfYdH8Gipec8Eeeu0xXdbba9frFj0=OqFfea0dXdd9vqai=hGuQ8kuc9pgc9s8qqaq=dirpe0xb9q8qiLsFr0=vr0=vr0dc8meaabaqaciaacaGaaeqabaqabeGadaaakeaacqWGJbWycqGHsislcqWGKbazcyGGSbaBcqGGUbGBdaWcaaqaaiabcIcaOiabiodaZiabdohaZjabgUcaRiabigdaXiabcMcaPiabgUcaRmaakaaabaGaeGynauJaem4Cam3aaWbaaSqabeaacqaIYaGmaaGccqGHRaWkcqaI2aGncqWGZbWCcqGHRaWkcqaIXaqmaSqabaaakeaacqaIYaGmcqWGZbWCdaahaaWcbeqaaiabikdaYaaaaaaaaa@462A@

**Figure 1:**
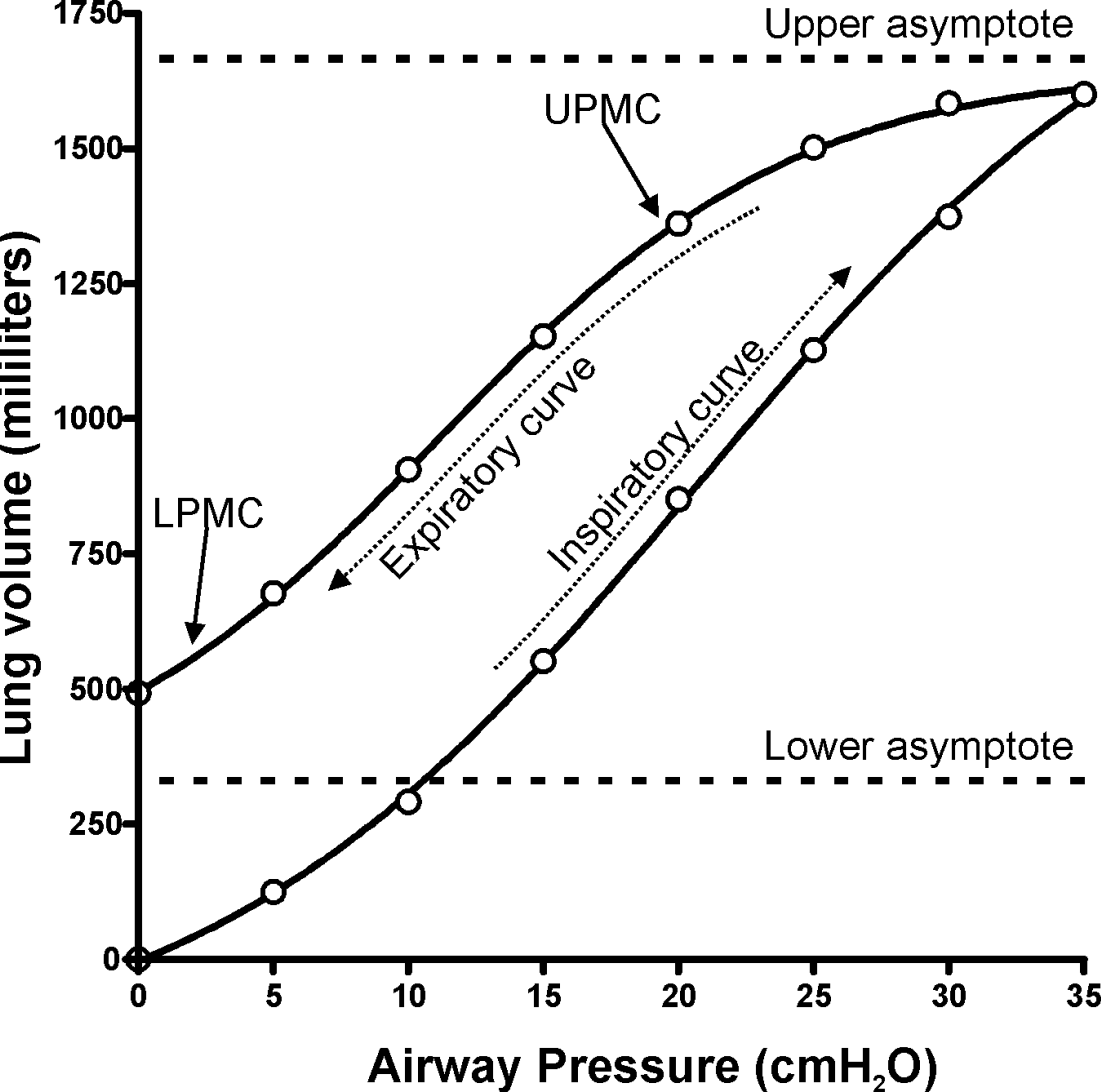
Pressure-Volume curve from one representative patient, showing the inspiratory and expiratory limbs of the curve and the parameters obtained after fitting the expiratory limb to a model. The fitted curve corresponds to model 1.

The total lung capacity was calculated as the upper asymptote of the curve. The maximum compliance was calculated as the maximal slope of the pressure-volume curve (as compliance is the first derivative of the pressure-volume curve, maximal compliance is the value where the second derivative equals zero). The inflection point and points of maximum curvature were calculated as the pressure values where the second and third derivatives of the model equal zero respectively. Due to the sigmoid nature of the models, there are two points of maximum curvature in each limb (the so-called "lower" and "upper" inflection points [LPMC and UPMC]). The general values of the calculated parameters are presented in table 1. Figure 1 shows a pressure-volume curve from one representative patient, including the parameters obtained after fitting to the equations.

The second derivative of model 3 is a transcendental equation, so a general term for pressure values when V" equals zero cannot be found. The values of inflection point and points of maximum curvature were numerically calculated using an iterative algorithm incorporated in a HP48 scientific calculator (Hewlett-Packard, Palo Alto, CA, USA).

### Statistics

Data pairs of pressure and volume were fitted to each model using non-linear regression with an iterative algorithm to minimize the sum of squared residuals (Levenberg-Marquardt method). The convergence criteria for this sum were fixed at 10^-8^.

Data are presented as arithmetic mean and standard deviation. The R^2 ^coeffients and the sums of squares (which represent the goodness of fit) were compared using an analysis of the variance [15], including the model and the curve limb as within-case factors. The values of the total lung capacity, maximum compliance and inflection points were compared using a one-way analysis of the variance. Post-hoc test were done when appropriate using Bonferroni's correction [16]. The within-case standard deviation was used to estimate the magnitude of the differences between the models [17]. A *p *value lower than 0.05 was considered significant. All calculations were done using SPSS 11.0 software (SPSS inc, Chicago, IL, USA).

## Results

### Goodness of fit

**Figure 2:**
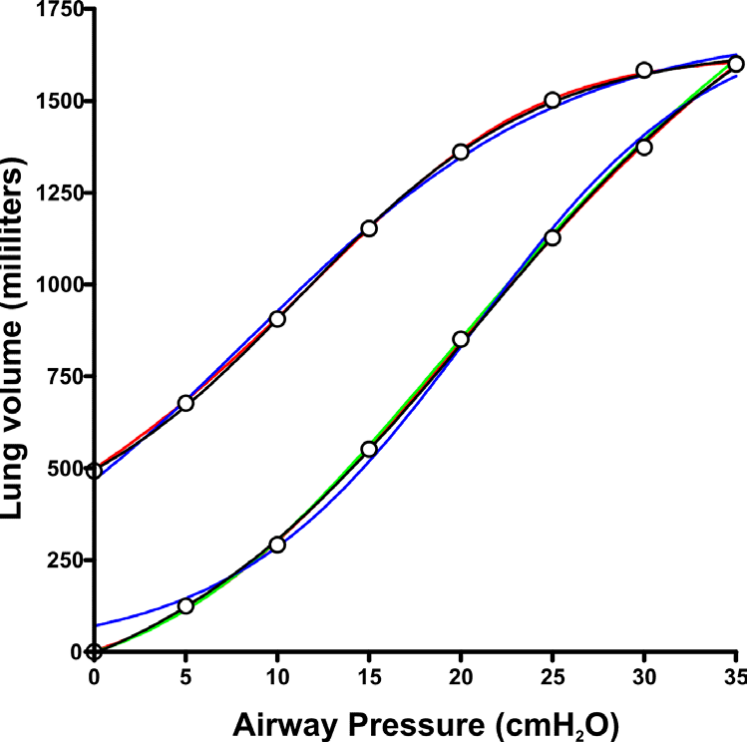
Pressure-Volume data from one patient, with the four curves corresponding to the mathematical models studied (black: model 1, blue: model 2, green: model 3, red: model 4).

All sets of data were fitted to the models, R^2 ^being always above 0.92. Model 4 provided the best fit (mean R^2 ^0.9985 [SD 0.0027]), followed by model 1 (mean R^2 ^0.9976 [SD 0.0035]), model 2 (mean R^2 ^0.9943 [SD 0.0061]) and model 3 (mean R^2 ^0.9849 [SD 0.0284]). These differences were significant (*p *= 0.011 in the ANOVA test, *p *< 0.01 in all post-hoc tests). The differences between inspiratory and expiratory limb fitting (*p *= 0.023 in the ANOVA) and the interaction between model and limb (*p *= 0.005) were also significant. Post-hoc tests revealed a significant difference between inspiratory and expiratory fitting only in model 3 (R^2 ^0.9982 [SD 0.0024] vs 0.9715 [SD 0.0497] for the inspiratory and expiratory limb respectively, *p *= 0.007). The same differences were found if the sums of squares instead of R^2 ^values were taken into account (data not shown). Figure 2 shows data from one patient with the four fitted curves.

### Upper asymptote and maximal compliances

The upper asymptotes yielded by model 3 were outside the expected limits in most of the cases (with values higher than 60 L), so this model was discarded in this comparison. There were significant differences in the predicted upper asymptote from the inspiratory curves (1836 ml [SD 680], 1647 ml [SD 532] and 2100 ml [SD 797] for the models 1, 2 and 4 respectively, *p *< 0.001 in the ANOVA and all post-hoc tests). The within-case standard deviation was 319.7 ml. However, the predicted upper asymptotes derived from expiratory curves were similar in the three models (1523 ml [SD 589], 1517 ml [SD 486] and 1536 ml [SD 577] ml for models 1, 2 and 4 respectively, *p *= 0.54). The within-case standard deviation was 117 ml.

There was a significant difference in the values of maximal inspiratory compliances in the four models (67 ml/cmH_2_O [SD 18], 72 ml/cmH_2_O [SD 20], 78 ml/cmH_2_O [SD 24], 65 ml/cmH_2_O [SD 18] for models 1 to 4 respectively, *p *= 0.019). Post-hoc tests revealed a significant difference between model 4 and models 1 and 2 (*p *= 0.01 in both cases). The within-case standard deviation was 7 ml/cmH_2_O. There were no differences in maximal expiratory compliances (59 ml/cmH_2_O [SD 21], 59 ml/cmH_2_O [SD 21], 57 ml/cmH_2_O [SD 19], 58 ml/cmH_2_O [SD 21] for models 1 to 4 respectively); *p *= 0.27). The within-case standard deviation was 4 ml/cmH_2_O.

### Points of maximum curvature

**Figure 3:**
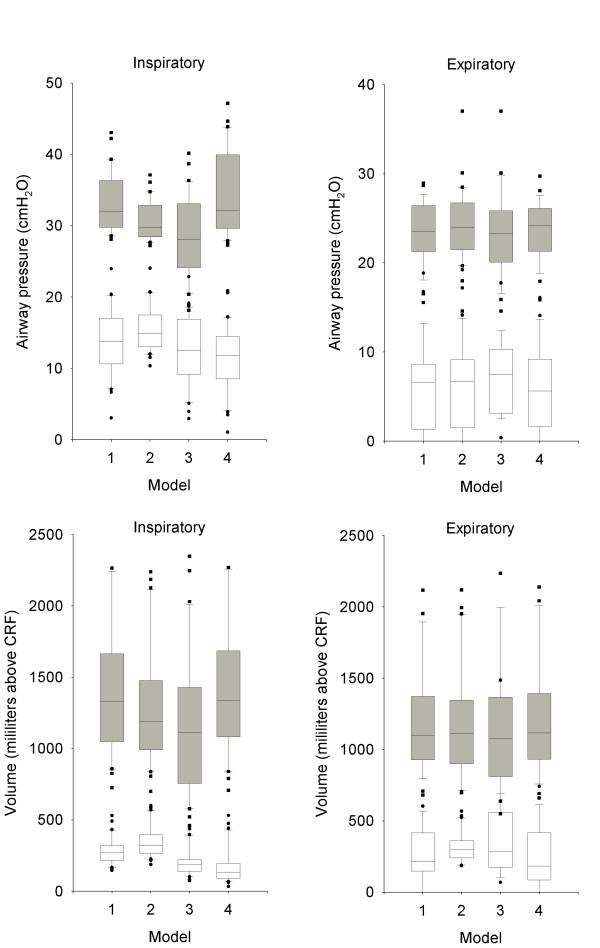
Values of pressure (upper row) and volume (lower row) at lower (white boxes, circles) and upper (grey boxes, squares) points of maximum curvature for each model. Significance levels are presented in text.

The models yielded different results for the maximum curvature points on the inspiratory limb of the pressure-volume curve, but not for those on the expiratory limb. In the same sense, lung volumes at these points were different only in the points from the inspiratory limb of the curve. All post-hoc test were significant (*p *< 0.05) except the comparison between inspiratory UPMCs derived from models 1 and 4 (*p *= 0.30 and 0.53 for pressure and volume respectively). These results are presented in figure 3. The within-case standard deviations for these data are presented in table 2.

**Table 2:** Within-case standard deviations of the values of pressure and volume at the points of maximum curvature.

	Pressures (cmH_2_O)		Volumes (ml)	
	LPMC	UPMC	LPMC	UPMC

Inspiratory limb	2.33	3.78	99	210
Expiratory limb	6.08	1.82	184	370

## Discussion

Our results show that the goodness of fit of these four mathematical models of the static pressure-volume curve is remarkably high. However, in contrast to our hypothesis, different models yield different results when applied in mechanically ventilated patients with acute lung injury. These differences are limited to the parameters derived from the inspiratory limb of the curve and could be relevant for the interpretation of the data.

### Utility of pressure-volume curves

The diagnostic or prognostic utility of the static pressure-volume cuve in acute lung injury has not been demonstrated. However, there is substantial interest in these curves as a tool to set mechanical ventilation in acute lung injury [3], with the goals of improving oxygenation and oxygen transport [18] and minimizing ventilator-induced lung injury [19]. The lower point of maximum curvature on the inspiratory limb of the curve has been used as a marker of optimal PEEP, and the upper as a marker of alveolar overstretching [20]. Three clinical trials have shown that a PEEP level selected according these points is related to a decrease in mortality [21,22] and a lower inflammatory response casued by mechanical ventilation [23]. Although there are experimental and clinical studies that challenge this interpretation of the pressure-volume curve [24], there is considerable interest in the use of lung mechanics to titrate therapy.

Some of the discrepancies between studies could be attributed to different approaches to determinate these points of maximum curvature. Harris et al [4] found substantial intra- and interobserver variability during the determination of the lower point of maximum curvature, especially with small data sets. The application of mathematical models could overcome these differences. Most studies have used the model described by Venegas and coworkers [4]. This consists in a sigmoid equation, symmetric to the inflection point of the curve. Other models have been proposed in an attempt to improve the flexibility to fit asymmetrical data [8,9], but some have not been tested in the clinical arena. Our results show that the fitting of data to different equations can lead to different values of the derived parameters. The magnitude of these differences is small in the majority of the results, but is substantial in the points of maximum curvature, which are the parameters more frequently used and studied. For instance, a within-case standard deviation of 2.33 cmH_2_O in the inspiratory LPMC implies that the difference between two models for the same subject is expected to be less than 6.45 cmH_2_O (2.77 times the standard deviation) for 95% of the pairs of observations. This is a considerable range of pressures if LPMC is used for PEEP setting. In a clinical trial in patients with acute lung injury, changes in PEEP were done using a table with steps of 2 cmH_2_O [25]. This shows that the differences between the models are above the range of pressures used in clinical practice.

Currently, some ventilators include tools to trace pressure-volume curves in an automatized fashion. Previously published results suggest that the analysis of these curves should not be done by eye fitting [4], so a mathematical analysis software must be included. Our results suggest that data about the mathematical model should be specified and taken into account in order to compare the results.

### Characteristics of the mathematical models

After the publication of a mathematical model by Venegas and coworkers (model 1 in this study), other models were published. Model 2 and 3 are based on the assumption that the lower asymptote is 0. This assumption is probably incorrect in some cases with air trapping, but it allows to reduce the number of parameters in the equation.

The other models were developed to overcome the symmetric nature of the original model. An asymmetric equation allows to improve the goodness of fit (in our study, the best result was obtained with model 4, although this model has 5 fitting parameters), but an increment in the number of parameters can lead to overfitting (an increase in the goodness-of-fit but a decrease in the significance and robustness of the parameters). In the range of pressures used in a clinical setting, symmetric models have shown an excellent fitting. However, in cases where data range reaches high values of pressure, an asymmetric model could be superior.

The symmetry of models 1 and 2 reflects a Gaussian distribution of airway opening and closing pressures. Tomographic studies in animals [7] and patients [26] with injured lungs have confirmed this distribution. As suggested by Harris and coworkers [4], and demonstrated later [13], pressure-volume curves should be considered as recruitment functions in acute lung injury, and the contribution of overstretching (which would be the prevalent phenomenon at lung volumes near the upper asymptote) to the shape of the curve is low. This could explain also that during deflation, as there is no ongoing recruitment, the four models yield similar results.

The main limitation of these models is that they are descriptive in nature, but without a mechanistic background. They come from the intention to fit the data and calculate parameters in a standardized fashion. However, they are not based in the behaviour of lung tissue during inflation and deflation. This descriptive nature could be one of the causes of the differences between the four models. Other could be related to the small number of data points used for fitting and the fact that what models yield as a concrete point is, from a biological point of view, a range of pressures where abrupt changes in lung mechanics occur. A mechanistic model, based in the mechanical properties of the injured lungs, should be developed to move beyond a simple description of the curve.

### Limitations of the study

Our study is subjected to some limitations:

1. It is a retrospective analysis of data. However, all the pressure-volume curves were performed using exactly the same methodology and the patients' characteristics in terms of course of the syndrome and severity were similar.

2. Limited pressure range. Only airway pressures up to 35 cmH_2_O were studied. This pressure level is probably associated to an incomplete aeration in some cases. A wider pressure range could have improved the results of the upper asymptote, especially in model 3, although the influence in the points of maximum curvature should be low. The range of pressures studied was selected according to clinical recommendations of airway pressure limitation [27].

3. Limited number of models: Other mathematical models were not considered. The selection was done because of the sigmoid nature of the equations.

## Conclusion

Sigmoid equations allow fitting pressure-volume data acquired from patients with acute lung injury with an excellent goodness-of-fit. However, there could be significant differences in the derived parameters from these models, specifically in the points of maximum curvature (which are used to set the PEEP level during mechanical ventilation). These differences should be taken into account when applying a model to a set of data or when comparing results from different studies.

## Abbreviations

CPAP: Continuous positive airway pressure.

LPMC: Lower point of maximum curvature.

UPMC: Upper point of maximum curvature.

PEEP: Positive end-expiratory pressure.

## Competing interests

The author(s) declare that they have no competing interests.

## Authors' contributions

GMA and FT designed the study. GMA acquired the data in patients and wrote the manuscript. GMA and EG made the mathematical and statistical analysis. All authors read and approved the manuscript.
